# DUSP1: Triple-Negative Breast Cancer and Therapeutic Potential

**DOI:** 10.3390/curroncol33020082

**Published:** 2026-01-30

**Authors:** Suryakant Niture, Dinesh Thotala, Jerry Jaboin, Danushka Seneviratne

**Affiliations:** 1 Department of Radiation Oncology, Oklahoma University Health Sciences Center, Oklahoma City, OK 73104, USA; 2 Stephenson Cancer Center, Oklahoma University, Oklahoma City, OK 73104, USA

**Keywords:** DUSP1, breast cancer, triple-negative breast cancer, therapy resistance

## Abstract

Triple-negative breast cancer (TNBC) is an aggressive breast cancer subtype characterized by high rates of recurrence, limited targeted treatment options, and frequent resistance to standard therapies. Dual-specificity protein phosphatase 1 (DUSP1), a stress-responsive regulator of mitogen-activated protein kinase (MAPK) signaling, has emerged as a context-dependent modulator of tumor progression and therapeutic response in TNBC. While reduced DUSP1 expression has been associated with aggressive tumor phenotypes and poor prognosis, accumulating evidence indicates that therapy-induced upregulation of DUSP1 can promote resistance to chemotherapy and radiotherapy by attenuating pro-apoptotic MAPK signaling and fostering immunosuppressive tumor microenvironment (TME). Emerging evidence highlights that DUSP1’s role is context-dependent on human cancers, including breast cancer (BC). This review synthesizes current evidence on DUSP1 biology in TNBC, with emphasis on its mechanistic involvement in chemotherapy resistance, radiation-induced immune modulation, and emerging implications for immunotherapy response.

## Simple Summary

Triple negative breast cancer (TNBC) is an aggressive and highly metastatic cancer that shows therapy resistance and poor prognosis in breast cancer patients. Due to the lack of expression of estrogen, progesterone, and HER2 receptors, hormonal therapy is unable to target TNBC and mostly relies on chemotherapy, radiotherapy, and immunotherapy as treatment options in the clinic. Downregulation of dual-specificity protein phosphatase 1 (DUSP1/MKP-1) in TNBC may promote TNBC cell proliferation and metastasis by activation of mitogen-activated protein kinases (MAPKs). In this brief review, we analyzed the expression profile of DUSP1 and discussed its correlation, significance, and therapeutic potential in TNBC with the current literature.

## 1. Introduction

Breast cancer (BC) is the second most common heterogeneous cancer among women globally, and in 2025, about 316,950 invasive BC cases will be estimated in the United States alone. One in eight women is diagnosed with BC in the United States, and overall, 5-year survival is about 91.7%. The worst 5-year survival rate, 78.4%, is observed in triple-negative breast cancer (TNBC) (American Cancer Society). TNBC accounts for approximately 15–20% of breast cancer cases and is associated with early recurrence, high metastatic potential, and poor overall survival (OS). BC is heterogeneous cancer and the heterogeneity of BC subtypes is mainly categorized as luminal A: hormone receptor HR+/HER2- (hormone receptor +/human epidermal growth factor 2-), luminal B: (HR+/HER2+ or HR+/HER2- with high Ki-67), HER2 enriched: (HR-/HER2+), and triple-negative: estrogen receptor (ER)-/progesterone receptor (PR)-/HER2-) [1]. The heterogeneity in BC also depends on the level of expression of specific genes, such as *ESR1*, *GATA3*, *HER2*, *ERBB2*, and is also associated with genetic mutations in *TP53* and *BRCA1/2*, and the tumor microenvironment (TME) around the TNBC tumor. Specifically, TNBC lacks the expression of ER, PR, and HER2 receptors [2,3], and the lack of these receptors and the mutation of genes are crucial for personalized treatment and predicting prognosis in TNBC patients [4,5].

Several strategies are applied to treat these BC-subtypes in the clinic that include chemotherapy, immunotherapy, surgery, and radiation therapy (RT) to improve loco-regional control of BC against metastasis and recurrence [6]. Importantly, each therapy interacts and interplays with other therapies; for example, chemotherapy sensitizes the BC tumor to death and increases the efficacy of RT, whereas RT improves systemic response to immunotherapy [7,8,9]. In most secondary metastatic BC cases, RT is used with adjuvant therapy, neoadjuvant therapy, and palliative therapy [10]. Since TNBC lacks HRs and HER2, targeting TNBC presents many more challenges.

Dual-specificity phosphatase 1 (DUSP1), also known as MAP kinase phosphatase 1, is a key negative regulator of MAPK signaling that dephosphorylates both threonine and tyrosine residues on activated MAPKs. Multiple studies have reported reduced DUSP1 expression in TNBC compared with other breast cancer subtypes, with low DUSP1 levels associated with aggressive tumor behavior, epithelial–mesenchymal transition, and poor clinical outcomes. In contrast, therapy-induced upregulation of DUSP1 has been implicated in resistance to cytotoxic chemotherapy and radiotherapy through regulation of MAPKs activities (JNK, ERK, and p38), modulation of inflammatory signaling pathways, and promotion of tumor cell survival. Importantly, these findings indicate that the role of DUSP1 in TNBC may be highly context-dependent and cannot be defined as uniformly tumor suppressive or tumor-promoting. Therefore, in this report, we specifically analyzed the regulatory role of dual-specificity protein phosphatase 1 (DUSP1/MKP-1), a MAPK phosphatase, in the therapy resistance of TNBC.

## 2. Triple-Negative Breast Cancer (TNBC)

The origin and risk of TNBC start from the mutations or changes in 19p13.1 locus on chromosome 19, the *MDM4* locus, and a unique pattern of germline mutations in the *BRCA1* gene [11,12]. Due to the high gene mutation rate in TNBC, especially in genes like *BRCA1* and *BRCA2*, it lacks a DNA repair mechanism, leading to increasing genomic instability and frequently losing ER, PR, and HER2 cell surface receptor expression [13,14]. Due to the lack of ER, PR, and HER2 expression, and increased expression of basal cytokeratin, TNBC adopts a basal-like (BL) cell profile, one of the BC subtypes. Although the BC subtype consists of 90% of other subtypes, luminal A (HR+/HER2-) 68%, luminal B (HR+/HER2+) 10%, HER2-overexpressing (HR-/HER2+) 4%, and unknown subtype 7%, whereas only 10 to 11% basal-like (HR-/HER2-) BC subtype population belongs to TNBC [15]. TNBC is further classified based on the percentage of basal-like cell profile, mesenchymal (M) like cell profile, and expression of luminal androgen receptor (LAR) cell profile [16,17]. TNBC consists of basal-like 1 (BL1) 35%, basal-like 2 (BL2) 22%, mesenchymal (M) 25%, luminal androgen receptor (LAR) 16% cell population [18].

TNBC is an aggressive breast cancer that mostly occurs in young women (~40 ages) as well as in older women, and a high incidence is reported in non-Hispanic black women [19]. Due to a lack of ER, PR, and HER2 receptors, treating TNBC with standard targeted therapies is difficult and mostly relies on chemotherapy, radiotherapy, and immunotherapy [20]. The five-year overall relative survival rate of patients with TNBC is worse compared with other BC subtypes. The five-year relative survival rate of the localized TNBC was 92.4%, regional TNBC was 76.5%, whereas distant TNBC was only 14.9% [21]. TNBC is an aggressive disease with a high rate of metastasis and recurrence, and the development of TNBC is associated with race, premenopausal status, and *BCRA1/2* inherited mutations; however, if diagnosed at stage I, II, or III, 80% to 90% of TNBC patients can potentially be cured. Treatment of TNBC depends on the stage of the cancer, ranging from Stage 0 (abnormal and non-invasive cells) to Stage IV (metastatic cancer cells) [22]. Generally, in the treatment plan at Stage I, chemotherapy followed by surgery (either a lumpectomy or mastectomy) and radiotherapy, TNBC at Stage II or III chemotherapy and immunotherapy, then surgery and possible radiotherapy may apply [22]. However, TNBC with Stage IV is not curable, but rather treatable. In such cases, molecular profiling, including immune cell-related PD-L1 expression, genomic testing (such as mutations in *BRCA1* or *BRCA2*), and therapy resistance tumor factors, as well as patient-specific clinical trials, are considered for further treatment options. Patients with metastatic TNBC (Stage IV) about 50% patients show an average survival of 1 ½ to 2 years [23]. Therefore, understanding the role of molecular factors that are associated with therapy resistance and TNBC recurrence is essential.

Recently, we reviewed the TNBC tumor microenvironment (TEM) and analyzed how RT modulated immune changes in TNBC-TEM [24]. We also analyzed the regulation and therapeutic significance of DUSP1 in various cancers [25]. In this brief report, we mainly focus on how DUSP1 modulates chemotherapy, radiotherapy, and immunotherapy sensitivity or resistance in TNBC.

## 3. DUSP1 Regulation in Breast Cancer (BC) Subtypes

DUSP1 is a dual-specificity phosphatase that dephosphorylates both threonine and tyrosine residues of the mitogen-activated protein kinases (MAPKs) and controls several key cellular processes, including cell proliferation, differentiation, apoptosis, stress responses, and drug resistance in cancer cells [25]. The activated MAPKs, through phosphorylation, induce resistance against chemotherapeutic drugs in several human cancers [25,26], and dysregulation of DUSP1 expression in BC tumors, particularly TNBC, disproportionately activates MAPKs and modulates the response of oncologic therapies.

**Figure 1:**
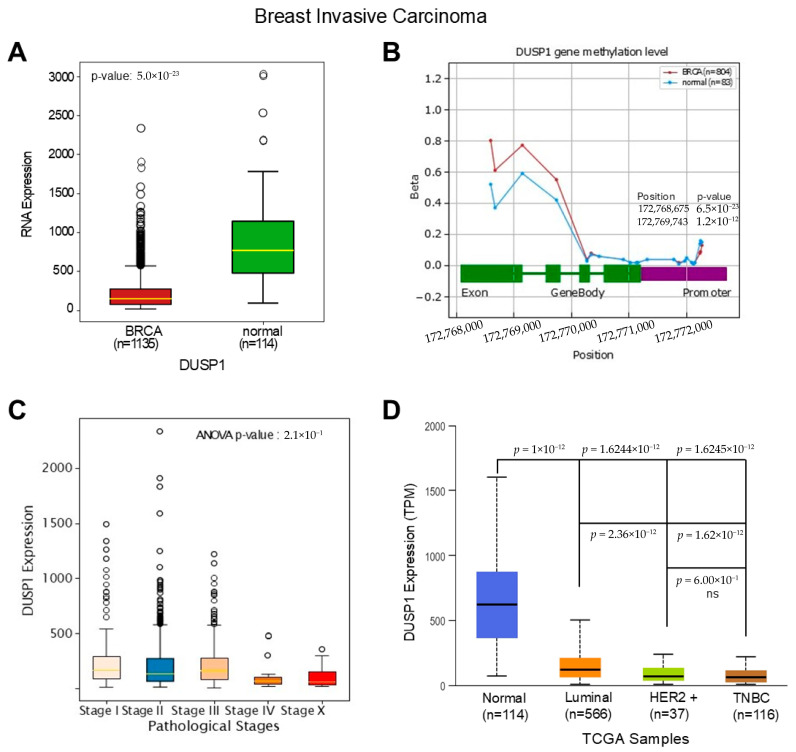
*DUSP1* expression in BC and TNBC. (**A**) The expression of *DUSP1* transcripts between BC tumor (*n* = 1135) and normal breast (*n* = 114) was analyzed from the TCGA data set, and the data were extracted from the OncoDB portal (https://oncodb.org/cgi-bin/expression_profile.cgi (accessed on 20 December 2025)) and presented. Statistical significance was determined by Student’s *t*-test, and the *p*-value was presented. (**B**) *DUSP1* gene methylation profile from BC tumor (*n* = 804) and normal breast (*n* = 83) was analyzed from the TCGA data set and OncoDB portal and presented. Statistical significance was determined by Student’s *t*-test, and significant *p*-values with positions were presented. (**C**) *DUSP1* expression levels in different Stages of BC were presented using the TCGA data set and OncoDB portal analysis. Statistical significance was determined by ANOVA, and the *p*-value was presented in the graph. (**D**) *DUSP1* expression levels in different BC subtypes were presented using the TCGA data set and UALCAN portal (https://ualcan.path.uab.edu/index.html (accessed on 12 December 2025)). Statistical significance was determined by Student’s *t*-test, and the *p*-values are presented between the two groups. *p*-value of <0.05 was considered statistically significant.

To understand the deregulatory role of DUSP1 in BC subtypes first, we analyzed the expression of DUSP1 transcripts in invasive BC tumors and compared them to normal breast tissues. TCGA data analysis suggests that *DUSP1* transcript expression is significantly downregulated in invasive BC tumors (*n* = 1135) compared to normal breast tissue (*n* = 114) (Figure 1A) (https://oncodb.org/cgi-bin/expression_profile.cgi (accessed on 20 December 2025)) [27]. The downregulation of *DUSP1* expression may occur due to the *DUSP1* gene methylation; however, as shown in Figure 1B, no significant change in methylation was found in the *DUSP1* promoter region in BC tumors compared with the normal breast cells’ *DUSP1* gene promoter. An increased methylation pattern was found in the exon and intron region of the *DUSP1* gene (primarily DNA methylation (5mC) and m6A) in BC tumor cells (Figure 1B). The promoter methylation often silences gene expression, whereas exon and intron methylation profoundly impacts gene expression by dysregulation of transcription, mRNA splicing, and mRNA stability [28,29]. The role of the exon and intron methylation on *DUSP1* transcript expression is not yet clear.

Because *DUSP1* expression is downregulated in invasive BC tumors, and to understand the significance of *DUSP1* downregulation, we further analyzed the expression levels of *DUSP1* in various Stages of BC tumors (Figure 1C). The expression of *DUSP1* transcripts decreased as BC pathological Stages increased, and at Stage IV presumably metastatic BC, the lowest expression of *DUSP1* was observed suggesting the reduction in DUSP1 expression promote advanced BC progression. Further, we analyzed the expression of *DUSP1* in BC subtypes. The *DUSP1* expression profile clearly indicates that, compared to normal breast, the *DUSP1* expression in Luminal, HER2+, and TNBC BC subtypes significantly decreased, and the lowest expression of *DUSP1* was observed in TNBC tumors (Figure 1D) (https://ualcan.path.uab.edu/index.html (accessed on 12 December 2025)) [30]. Moreover, *DUSP1* expression was significantly downregulated in HER2+ and TNBC compared with luminal BC subtypes, whereas there was no significant change in *DUSP1* expression observed between HER2+ and TNBC BC tumors (Figure 1D).

**Figure 2:**
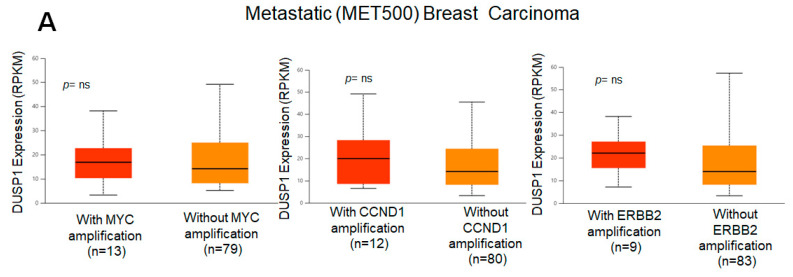

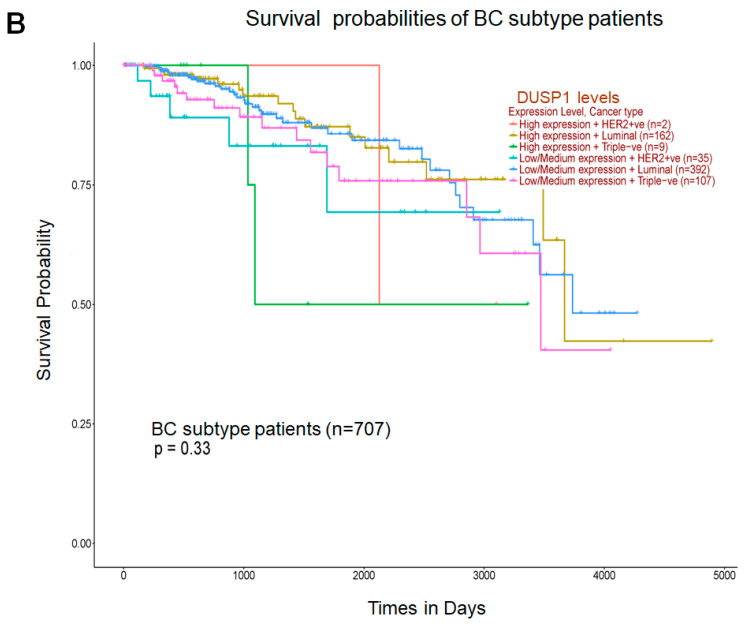
*DUSP1* expression and BC subtype patient survival. (**A**) Co-relation of *DUSP1* expression with and without *MYC*, *CCND1,* and *ERBB2* in metastatic (MET500) BC patients was presented. (**B**) Survival probabilities of BC-subtype patients with low and high *DUSP1* expression were presented from the UALCAN portal. (https://ualcan.path.uab.edu/index.html (accessed on 12 December 2025)). Statistical significance between the groups was determined by the *t*-test, and the *p*-values are presented. A *p*-value of <0.05 was considered statistically significant.

Because *DUSP1* expression was downregulated in the highly aggressive and metastatic TNBC tumor, we analyzed the correlation of DUSP1 expression with three important genes that participate in BC aggressiveness and metastasis, such as *MYC* [31], *CCND1* [32], and *ERBB2* (*HRE2*) [33]. TCGA correlation analysis suggests that the expression of *DUSP1* shows a weak positive linear relationship (R = 0.2) with *MYC* gene expression and a weak and no relationship with *CCND1* or *ERBB2* expression in BC tumors [34]. Interestingly, in metastatic BC tumors, the expressions of *MYC*, *CCND1*, and *ERBB2*, and the expression of *DUSP1* did not significantly change, suggesting that *DUSP1* expression may contribute to BC metastasis like *MYC*, *CCND1*, and *HER2* expression (Figure 2A). The role of DUSP1 expression and cell metastasis needs further investigation. Finally, we analyzed the survival probabilities of BC subtype patients associated with DUSP1 expression (Figure 2B). Although the patient size is small, as indicated in Figure 2B, TNBC patients with low DUSP1 expression (*n* = 107) show a better prognosis but lower survival compared to high DUSP1-expressing TNBC patients (*n* = 9). Overall, these data suggest that DUSP1 is a key regulator of survival in BC patients, particularly in those with advanced and metastatic TNBC.

In the following sections, we will review the interference, significance, and therapeutic modulatory role of DUSP1 in highly metastatic TNBC.

## 4. DUSP1 Regulation and Its Role in TNBC Chemotherapy

Although the low expression of DUSP1 is reported in TNBC, several studies suggest that DUSP1 plays a crucial role in modulating TNBC progression. He et al. utilized the microarray technique to characterize the molecular gene profiling between TNBC and non-TNBC subclasses [35]. Functional annotation for these differentially expressed genes (DEGs) and validation of gene expression clearly suggest that the expression of *DUSP1* is downregulated in several TNBC cell lines compared to HR-positive BC cells [35]. The protein–protein interaction network analysis suggests that DUSP1 may be considered as a potential therapeutic target for TNBC treatment [35]. Several factors contribute to the regulation of *DUSP1* in TNBC. For example, the cause of low expression of *DUSP1* in TNBC is due to the *DUSP1* gene methylation in ER/PR-negative BC. A high frequency of *DUSP1* methylation was found in TNBC patients, not only in tumor DNA samples but also in the peripheral blood leukocyte (PBL) DNA [36]. The increased *DUSP1* methylation in TNBC is associated with several diet factors (fruit and soybean intake), irregular menstruation, environmental factors, and the ER/PR status in the BC tumor [36]. Since circRNAs bind with microRNA and prevent them from inhibiting their target messenger RNAs (mRNAs) and regulate gene expression [37]. A recent study suggests that the aggressiveness of TNBC can be controlled by circular DUSP1 (circDUSP1) RNA in TNBC. Jian et al. identified and analyzed circDUSP1 RNA, which was significantly downregulated in TNBC cells, and overexpression of circDUSP1 RNA inhibits TNBC cell proliferation, migration, invasion in vitro, and TNBC tumor growth in vivo [38]. Mechanistically, circDUSP1 RNA binds with microRNA-429 to relieve its repression of the tumor suppressor Deleted in Liver Cancer (*DLC1*) gene, thus circDUSP1 RNA acts as a molecular sponge for microRNA-429 to upregulate *DLC1*, which then suppresses TNBC growth.

The high expression of glucocorticoid receptor (GR) in the early stage of TNBC correlates with chemotherapy resistance and increased recurrence. An earlier study revealed that the GR antagonist mifepristone alone does not show a significant effect on TNBC cell viability or clonogenicity; however, with mifepristone and chemotherapeutic drugs, dexamethasone/paclitaxel treatment increased cytotoxicity and apoptosis. Mechanistically, mifepristone antagonized GR-induced *SGK1* and *DUSP1* gene expression while significantly increasing tumor shrinkage in paclitaxel-induced MDA-MB-231 TNBC xenograft in vivo, suggesting that GR antagonist mifepristone may be useful for suppression of chemotherapy-resistant GR + TNBC [39]. On the other hand, high levels of β2-adrenergic receptor expression were observed in TNBC, which directly modulate the activity of extracellular signal-regulated kinase (ERK) 1/2. Exposure of β2-adrenergic receptor agonists increased dephosphorylation of basal pERK1/2 in TNBC (MDA-MB-231 and MDA-MB-468) cells. Interestingly, β2-adrenergic receptor activation by agonists increased DUSP1 expression and the active form of PP1, and the inactivation of DUSP1 by (E)-2-benzylidene-3-(cyclohexylamino)-2,3-dihydro-1H-inden-1-one (BCI) and PP1/PP2 by calyculin A or downregulation of DUSP1 and PP1 decreased the β2-adrenergic receptor-mediated dephosphorylation of ERK1/2 suggesting DUSP1 and PP1 activity required for dephosphorylation of ERK1/2. Indeed, the study further suggests that the use of β2-adrenergic blockers may be useful for TNBC patient treatment via DUSP1 regulation [40]. Basal-like breast cancer (BLBC) and TNBC show significant biological overlaps (around 60–90%), and BLBCs show resistance to endocrinotherapy or hormone therapy. Recent studies suggest that paclitaxel (PTX) resistance in TNBC MDA-MB-231 cells shows downregulation of DUSP1 and DUSP5 and is negatively associated with higher histological tumor grade in BC patients. Interestingly, the study revealed that low expression of DUSP1 was found in HER2+ basal-like cells, whereas DUSP5 low expression was mainly observed in basal-like cells compared with other subtypes, suggesting that not low DUSP1 expression but loss of DUSP5 expression was associated with PTX resistance and poor survival of BLBC patients, thus restoring DUSP5 level may improve therapeutic potency of BLBC [41]. Since the expression of DUSP1 is downregulated in TNBC. Several compounds may activate DUSP1 expression and activity; for example, an organic c-glycosyl compound, aurovertin B (AVB), exposure to TNBC cells upregulates DUSP1 expression and, by interacting with DUSP1, AVB-DUSP1 activates activating transcription factor 3 (ATF3) and subsequently suppresses TNBC metastasis [42].

As we indicated earlier, the role of DUSP1 is context-dependent in cancers [25]. Several studies analyzed the effect of DUSP1 on MAPK signaling under the influence of chemotherapeutic drugs. In human mammary epithelial cells A1N4-*myc*, BC BT-474 triple-positive, and TNBC MDA-MB-231 cells, a transient and stable overexpression of DUSP1 and treatment of mechlorethamine (DNA alkylating agents), doxorubicin (an anthracycline drug), and paclitaxel (microtubule inhibitor) show high suppression of JNK1 phosphorylation in MDA-MB-231 and BT-474 cells, decreased DNA fragmentation, apoptosis, and increased chemoresistance by increasing cell proliferation [43]. In contrast, DUSP1 knockdown (siRNA) or inhibition (SD-282 inhibitor) followed by single or combined exposure to doxorubicin and mechlorethamine increased drug sensitivity through JNK activation, suggesting that targeting DUSP1 may overcome the drug resistance and promote chemo-sensitization [43]. Similarly, the reduction in DUSP1 by using CRISPR/Cas9 and siRNA or the inhibition of DUSP1 by BCI exposure suppressed cell proliferation, migration, and tumor growth in the TNBC xenograft model [44]. Downregulation of DUSP1 increased the phosphorylation of p38 and JNK, but not ERK1/2, and increased anticancer response induced by cisplatin. Importantly, cisplatin exposure increased p38 phosphorylation by reducing DUSP1 expression in TNBC cells [44]. Collectively, these in vitro studies suggest that targeting or downregulation of DUSP1 increases the efficacy of anticancer drugs.

Furthermore, epithelial-to-mesenchymal transition (EMT) plays a crucial role in chemotherapy resistance in TNBC, enabling TNBC cells to acquire a stem-like appearance, thereby promoting invasiveness and chemoresistance [45]. The DUSP family members play a crucial role in EMT and cancer stem cell (CSC) regulation in BC and TNBC [46]. In BC cells, immunocytochemical data suggest that DUSP1, DUSP4, and DUSP6 differentially co-exist with enhancer and permissive active histone post-translational modifications and modulation of EMT and CSC-like transitions. Importantly, MDA-MB-231 cells, DUSP1, and DUSP4 were mostly localized in the nucleus, while DUSP6 showed strong cytoplasmic localization. In TNBC MDA-MB-231 cells, DUSP1 showed high levels of co-localization with the active promoter marks H3K9me1 (PCC = 0.65) and H3K4me3 (PCC = 0.72), indicating DUSP1 plays distinct roles in EMT gene regulation in TNBC cells. The study further revealed that knockdown of DUSP1, DUSP4, and DUSP6 regulates the formation of CD44^hi^/CD24^lo^/EpCAM^+^ breast CSCs. Interestingly, DUSP1 knockdown reduces CSC formation, while DUSP4 and DUSP6 knockdown enhance CSC formation in BC [46]. In addition, in TNBC cells, the lack of tumor suppressive microRNA-200 expression and loss of 3’UTR may increase DUSP1 expression and promote EMT, and miR-200b-3p overexpression represses EMT [47]. Yuan et al. have identified differentially expressed ferroptosis-related genes in TNBC patients and found that seven genes, such as *DUSP1*, *STEAP3*, *CISD1*, *HMOX1*, *CA9*, *TAZ*, and *HBA1*, were associated with the overall survival (OS) of TNBC patients [48]. High expression of S*TEAP3* was associated with overall survival rates of TNBC patients, although the role of DUSP1 is not clear.

Indeed, DUSP1 is a negative regulator of the MAPK pathway to maintain cell proliferation and survival; however, in TNBC, downregulation of DUSP leads to aberrant expression of several pathways, and the exact mechanisms by which DUSP1 modulates EMT, metastasis, and chemotherapy escape remain unknown so far.

## 5. DUSP1’s Role in TNBC Radiotherapy

Radiotherapy not only kills or shrinks the cancerous tumor but also plays a crucial role in cancer treatment to enhance chemotherapy or immunotherapy efficacy [49]. Several cancer tumorigenic factors can cause radioresistance; for example, activation of transcription factors and signaling pathways (Wnt, Notch, STAT, PI3K/AKT, NF-κB, and Hedgehog), induction of DNA repair mechanisms, EMT, tumor-derived production of cancer stem cells (CSCs), and dynamics of tumor microenvironment (TME), which prompts stemness in the tumor [50]. Early studies indicate that DUSP1 and MAPKs play a role in redioresistance [51]. For example, overexpression of DUSP1 inactivates stress-activated protein kinase (SAPK) and MAPK (p38) and protects human monocyte-like histiocytic lymphoma cells against UV-induced apoptosis [52], whereas mouse embryonic fibroblasts deficient in *DUSP1* show increased UV sensitivity due to activation of MAPKs (p38α and JNK) [53], suggesting DUSP1 modulates radiation response in tumor cells. In BC MCF7 cells and TNBC MDA-MB-231 cells, radiation exposure (2 h) induced DUSP1 expression and translocated into mitochondria, by interacting with JNK, reduced phosphorylated active forms of JNK kinase [54]. To analyze the radiosensitivity and radioresistance between BC cell-subtypes, the authors have utilized MCF7 wt (HER2-low), SKBR3 (HER2-positive), MCF7/C6 (HER2-overexpressing), MDA-MB-231 (HER2-negative), and HER2-positive BCSCs (HER2^+^/CD44^+^/CD24^−/low^) cells. The silencing of DUSP1 expression and radiation exposure clearly indicated that HER2-positive breast cancer cells were more sensitive to radiation compared with HER2-low/negative cell lines. Clonogenic survival assay suggests that knocking down DUSP1 in HER2-overexpressing SKBR3 (ER-/PR-) cells and TNBC MDA-MB-231 cells inhibit 65% and 20% cell colony formation, respectively. However, MDA-MB-231 cells show high radiosensitivity after DUSP1 knockdown compared to SKBR3 cells, suggesting that DUSP1 is important for HER2-positive breast cancer cells for survival compared to HER2-negative or TNBC MDA-MB-231cells [54]. The author further suggests that combined inhibition of DUSP1 and HER2 may enhance radiosensitivity in HER2-positive BC cells [55].

Radioresistance in BC is common [24,56] because of BC tumor heterogeneity/subtypes, genetic mutation, presence of CSCs [57], impaired DNA repair mechanisms, dysregulated oncogenic signaling, and cell cycle traits [58]. Recently, in our laboratory, we analyzed the impact of radiation therapy (RT) on targeted gene expression (NanoString nCounter approach) and comparatively analyzed the differential gene expression (DGE) profile between biopsy (pre-RT) vs. surgical (post-RT) BC tumors. Our data suggest RT induced expression of *DUSP1* and macrophage M2-associated *CD163* in BC tumors. Importantly, DUSP1 expression is not only found in tumor cells but also in immune cells, such as macrophages, and is also released in the serum upon cell damage or during apoptosis [59,60,61], and serum circulating levels of DUSP1 can be measured with ELISA [61] for monitoring any correlation with patient prognosis. In addition, our data suggest that RT also enhanced residual cancer burden (RCB) in BC tumors, thus creating an immunosuppressive TME. Because DUSP1 plays a complex role in BC tumor growth, metastasis, therapy resistance, and context-dependent function, currently, in our laboratory, we are analyzing the impact of RT on tumor and serum-associated DUSP1expression in patients with BC and TNBC tumors with various pathology grades, TNM staging, and HR status, to understand the role of DUSP1 in radiation efficiency and radioresistance.

## 6. DUSP1’s Role in Immunotherapy

**Figure 3:**
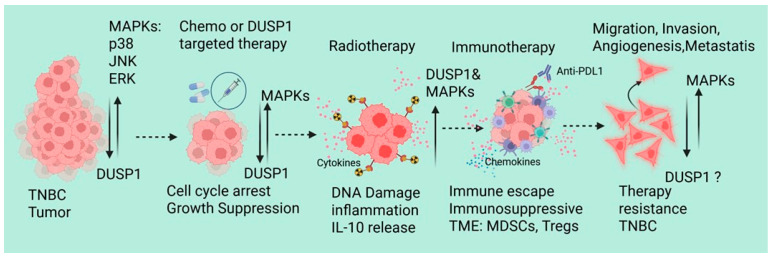
A schematic represents the possible role of DUSP1 in TNBC during chemotherapy, RT, and immunotherapy. Created in BioRender. Seneviratne, D. (2026) https://BioRender.com/ll2fa9d (accessed on 10 December 2025).

Since DUSP1 modulates chemotherapy and radiotherapy resistance in TNBC, recent emerging evidence also supports that the dysregulation of DUSP1 in TNBC affects immune response and immunotherapy resistance. Lui et al. identified and analyzed the single-cell RNA-sequencing data of peripheral blood mononuclear cells from 22 TNBC patients, including 11 pretreatment (PR), 7 stable disease (SD), and 4 partial response (PR) patients who received paclitaxel in combination with anti-PD-L1 (atezolizumab) immunotherapy [62]. RNA-sequencing data suggest that there is a significant increase in the myeloid cells in the PR and SD groups, also show an increase in the population of C8: CD8+ NKT cells and C2: classical monocytes in the immunotherapy response group, whereas dendritic cells and non-classical monocytes derived from classical monocytes were decreased. In CD8+ NKT cells and classical monocytes from these treatment groups shows a downregulated ferroptosis-related gene (DDIT4) in CD8+ NKT cells, which can inhibit the activity or proliferation of CD8+ NKT cells. In classical monocytes, the expressions of *DUSP1*, *FTL*, *BID*, *CD44*, *SLC2A3*, *DDIT4*, and *JUN* genes were significantly associated with the immune response. Further analysis of mutation profiles of differentially expressed FRGs (TCGA transcriptomic data) suggests that in TNBC patients, an increased copy number of *DUSP1* and *DDIT4* genes, missense mutations in the *DUSP1* gene, and nonsense mutations in the *DDIT4* gene. Interestingly, the *DUSP1* mutation shows worse prognoses for TNBC patients, indicating that *DUSP1* may play a crucial role in immunotherapy resistance in TNBC [62]. During neoadjuvant chemotherapy (NAC), patients with TNBC show distinct immune cell-associated gene expression signatures [63]. In the NAC responder, lymphocyte and monocyte populations consistently produce stress response factors such as *FOSB*, *JUN*, and *JUNB*. In monocyte clusters, *DUSP1* and *DUSP2* showed similar expression patterns in both patients (responder and non-responder). In T/NK cells, however, *DUSP1* and *DUSP2* remained stable in the responder patient but increased by day 21 in the nonresponder. The exact role of the differential expression *DUSP1* in monocyte clusters and T/NK cells, and immune modulation and NAC resistance, remains unknown [63]. Metastatic BC and single-cell transcriptomes of circulating immune cells suggest upregulation of *HLA-DQA1*, a B-cell-specific gene marker that is associated with antigen presentation. However, DUSP1, CD69, and FOS inflammatory markers expression is downregulated, indicating a less active inflammatory signature in metastatic BC tumors [64]. It will be interesting to investigate the DUSP’s role in immunotherapy modulation and resistance in TNBC. Although TNBC-associated molecular mechanisms, tumor microenvironment, and therapeutic implications have been reviewed recently [65], we also represented the possible role of DUSP1 in TNBC during therapies (Figure 3).

## 7. Conclusions

DUSP1 functions as a central, context-dependent regulator of therapeutic response in triple-negative breast cancer, integrating stress-activated MAPK signaling with tumor cell survival and immune modulation across chemotherapy, radiotherapy, and immunotherapy. Rather than acting as a uniformly tumor suppressive or tumorigenic factor, DUSP1 exerts divergent effects determined by cellular compartment, treatment context, dominant MAPK axis, and disease stage. Activation of MAPKs drives chemotherapy resistance against doxorubicin, paclitaxel, and cisplatin drugs; however, the impact of these drugs in acquiring chemotherapy resistance in TNBC was not known. Radiation therapy may activate/stabilize DUSP1, as well as MAPKs independently, and activation of anti-inflammatory signaling may attract immunosuppressive MDSCs and macrophages into the tumor microenvironment in order to boost TNBC cell aggressiveness and metastasis. Indeed, for better management of TNBC clinically, increasing TNBC patients’ clinical trials, examining the context-dependent mechanisms of DUSP1, and developing a selective therapeutic approach will be the first steps in pursuing DUSP1-based therapy for TNBC patients.

## Data Availability

No new data were generated in this study.

## Abbreviations

The following abbreviations are used in this manuscript:

DUSP1	Dual-specificity protein phosphatase 1
MAPKs	Mitogen-activated protein kinases
EMT	Epithelial–mesenchymal transition
TNBC	Triple-negative breast cancer
HR+	Hormone receptor positive
BRCA1/2	Breast Cancer gene ½
HER2	Human epidermal growth factor receptor 2
TP53	Tumor protein 53
RT	Radiation therapy
BC subtypes	Brest cancer subtypes
TME	Tumor microenvironment
CCND1	Cyclin D1 gene
circRNAs	Circular RNAs
BCI	(E)-2-benzylidene-3-(cyclohexylamino)-2,3-dihydro-1H-inden-1-one
BLBC	Basal-like breast cancer
PTX	Paclitaxel
CSCs	Cancer stem cells
